# Placental acute inflammation infiltrates and pregnancy outcomes: a retrospective cohort study

**DOI:** 10.1038/s41598-021-03655-4

**Published:** 2021-12-17

**Authors:** Maria Orsaria, Stefania Liviero, Emma Rossetti, Carla Pittini, Lorenza Driul, Ambrogio P. Londero, Laura Mariuzzi

**Affiliations:** 1 Institute of Pathology, DAME, University Hospital of Udine, 33100 Udine, UD Italy; 2 Clinic of Obstetrics and Gynecology, DAME, Academic Hospital of Udine, University Hospital of Udine, Piazza Santa Maria della Misericordia, 15, 33100 Udine, UD Italy; 3 Obstetrics and Gynecology, Hospital of Bressanone, 39042 Bressanone, BZ Italy; 4 Unit of Neonatology, University Hospital of Udine, 33100 Udine, UD Italy; 5 Ennergi Research, 33050 Lestizza, UD Italy

**Keywords:** Predictive markers, Prognostic markers, Medical research

## Abstract

Chorioamnionitis can be either an infection or a sterile inflammation. This study aims to analyze the prevalence of acute inflammatory lesions of the placenta, the association with a positive result of the microbiological examination, and the fetal-maternal outcomes. This retrospective study considered all single, consecutive pregnancies and their placental pathological examination during 2014–2017. The evidence of funisitis, chorionic vasculitis, and chorioamnionitis was assessed by a pathologist, including stage and grade. Moreover, maternal fever, placental microbiological examination, and neonatal outcomes were also recorded. Among the 5910 pregnancies in the considered period, 1770 had a placental pathological examination, and 358 (6.06%) had acute placental inflammation. Microbiological examination was performed in 125 cases, revealing 64 cases with a positive microbiological outcome. In the presence of acute placental inflammation, there was a higher rate of neonatal cardiopulmonary resuscitation, admission to neonatal intensive care unit, and postnatal death of the newborn. Multivariate analysis inferred that acute inflammation of membranes was a risk factor for neonatal cardiopulmonary resuscitation (OR 2.12; CI.95 1.36–3.31; p < 0.05), acute funisitis was a risk factor for admission to intensive neonatal care unit (OR 3.2; CI.95 1.67–6.12; p < 0.05), and chorionic vasculitis was a risk factor for postnatal death of the newborn (OR 5.38; CI.95 1.37–21.06; p < 0.05). The prevalence of chorioamnionitis was 6.06%, and about half of the cases were sterile inflammation. Chorioamnionitis was associated with higher rates of adverse fetal and neonatal outcomes; in particular, chorionic vasculitis was a risk factor for postnatal death.

## Introduction

The term chorioamnionitis refers to the acute sterile inflammation or the infection of placental membranes, chorionic plate, umbilical cord, fetus, or amniotic fluid^1^. Clinical chorioamnionitis is traditionally characterized by maternal and fetal tachycardia, maternal fever and leukocytosis, purulent or malodorous amniotic fluid, and uterine tenderness. Meanwhile, histologic chorioamnionitis is often subclinical and detected only after histopathologic examination. In histologic chorioamnionitis, the microscopic exam reveals acute inflammatory lesions of the placenta characterized by the infiltration of neutrophils into the chorionic plate, placental membranes, or umbilical cord^2–4^. In most cases, the inflammation results from ascending bacterial infection from the lower genital tract, which is demonstrated through the microbiological examination of the fetal adnexa^4^. However, a "Sterile intraamniotic inflammation" can occur in the absence of demonstrable microorganisms^5,6^. The frequency of chorioamnionitis varies according to diagnostic criteria, specific risk factors, and gestational age^7,8^. It is generally diagnosed in 1–10% of pregnancies, but preterm pregnancies show higher frequencies^3,7^. Chorioamnionitis is associated with significant adverse maternal-neonatal outcomes. Maternal consequences include postpartum infection, sepsis, and multiorgan damage. On the neonatal side, it can lead to preterm birth, stillbirth, sepsis, neonatal death, chronic pulmonary disease, cerebral palsy, and neurodevelopmental impairment^4,9^. A better understanding of the epidemiology and the mechanisms that determine this fearsome pathology is likely to impact significantly maternal and infant health. Thus, this study aims to determine the prevalence of acute placental inflammation, its association with microbiological positivity, and maternal–fetal outcomes.

## Methods

This retrospective chart review study considered all single, consecutive pregnancies delivered during the 2014–2017 period in our Academic Hospital (tertiary referral center). After 22 weeks and 6 days of gestation, all pregnancies whose placenta was submitted to pathological examination for maternal, placental, or fetal indications were included in the study. Multiple gestations were excluded.

Clinical data were gathered from pregnancy files, and the following data were considered: maternal age, nulliparity, spontaneous conception vs. assisted conception treatments (intrauterine insemination (IUI), in vitro fertilization/intracytoplasmic sperm injection (IVF/ICSI)), gestational age at delivery, ethnicity, hospitalization during pregnancy, pregnancy-related hypertensive disorders (PRHDs), mode of onset of labor, mode of delivery, and maternal fever. The following neonatal data were appraised: Apgar score at the first and fifth minute, neonatal weight at delivery, neonatal weight MoM, placental weight, small for gestational age (SGA), large for gestational age (LGA), intrauterine growth restriction (IUGR), neonatal cardiopulmonary resuscitation, neonatal intensive care unit (NICU) admission, neonatal outcome (alive, stillbirth, postnatal death).

Gestational age was determined by withholding the last known menstrual period from the date of birth of infants, was confirmed by ultrasound examination during the first and second trimester, and was expressed in weeks. Preterm delivery was set as birth before 36 weeks and 6 days of gestation. As previously described, ethnicity was considered stratifying the population by macro-regions and cultural backgrounds: Italy and Western Europe; Eastern Europe; Sub-Saharan Africa; Arabian Countries that included North Africa, Southwest and South Asia (excluded Nepal, Bangladesh, and Bhutan); Asia (that included Nepal, Bangladesh, Bhutan, North, Central, East, and Southeast Asia); other countries (America and Oceania)^10,11^. Hypertension was designated as high systolic blood pressure (≥ 140 mmHg) or high diastolic blood pressure (≥ of 90 mmHg)^12^. The following items were grouped as PRHDs: eclampsia, pre-eclampsia, pre-eclampsia superimposed on chronic hypertension, and gestational hypertension^10,13^. As previously defined, labor could be spontaneous, induced, or absent, and delivery could be spontaneous, operative vaginal delivery, or by cesarean section^10^. Maternal fever was defined as a temperature > 38 °C (100.4°F)^1^. IUGR was fixed as the sonographic finding of fetal weight below the tenth percentile of expected weight for gestational age (using Hadlock formula) associated with an increased PI in the umbilical arteries (greater than two standard deviations) or an estimated fetal weight below the third percentile^14^. In this study, SGA and LGA were defined as neonatal weight under the 10th and over the 90th percentiles for gestational age, respectively^14^. Neonatal death that occurs at less than 28 days of age was defined as perinatal death^10,15^.

### Placental examination

According to the internal protocol, which expands the criteria given by the College of American Pathologists, the indications for pathologic examination were: a maternal history of previous miscarriages or intrauterine fetal demise (IUFD); maternal pathologies (e.g., hypertension, diabetes mellitus, drug abuse), preterm deliveries, fetal pathologies in the current pregnancy (e.g., IUGR, oligohydramnios, polyhydramnios, hydrops or fetal malformations), gross placental abnormalities, fetal distress during labor, need of neonatal cardiopulmonary resuscitation, maternal fever in labor or suspected chorioamnionitis^16^.

**Figure 1:**
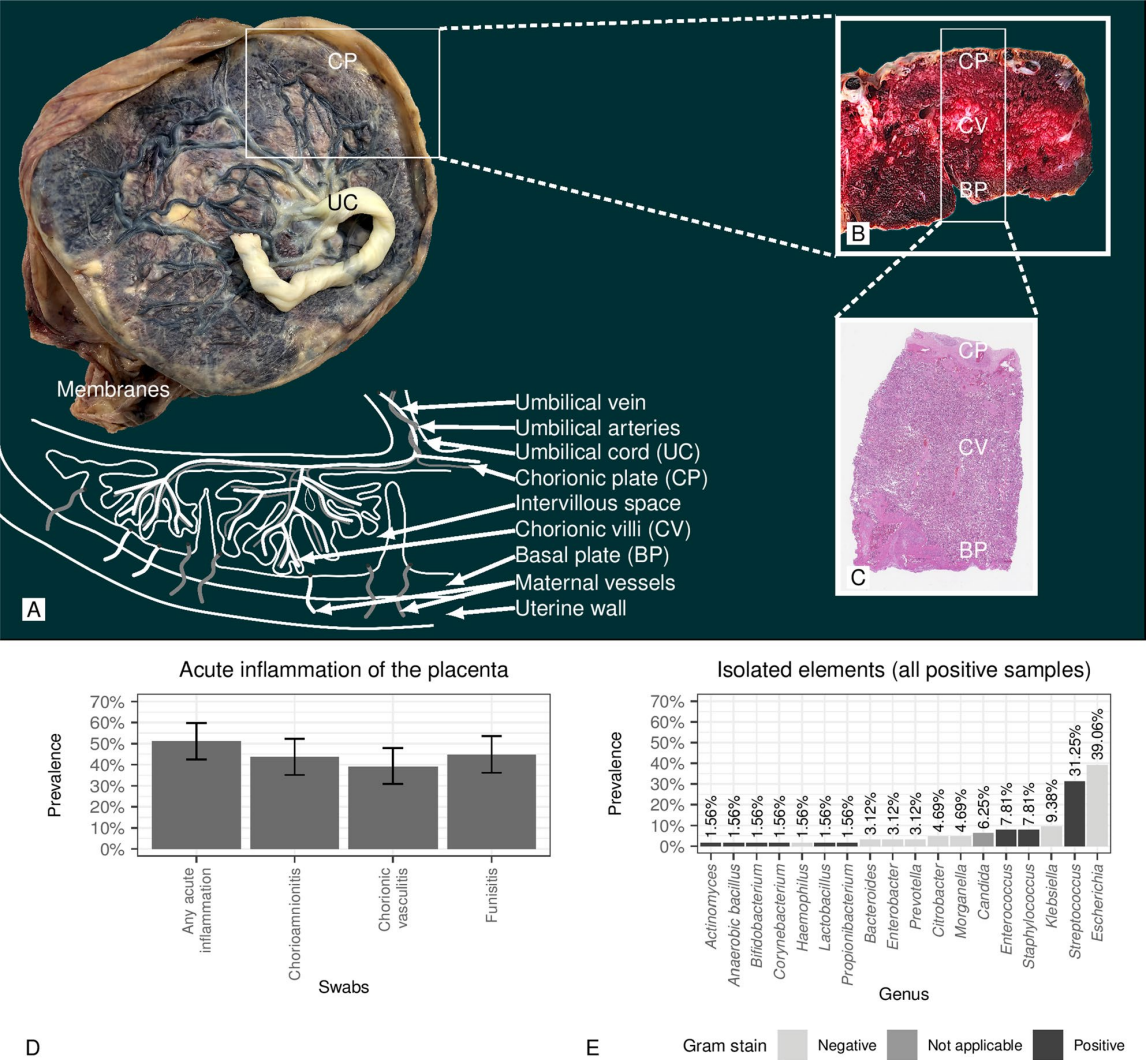
**(A–C)** show gross and microscopic figures from placenta (acronyms: *UC*  umbilical cord, *CP*  chorionic plate, *CV*  chorionic villi, *BP*  basal plate [maternal side of the placenta]). **(A)** Illustrates the whole placenta with membranes and umbilical cord. **(B)** Gross figure that is taken from through placenta section. **(C)** Whole placenta thickness microscopic figure. **(D)** Prevalence of positive swabs in cases of acute inflammation of the placenta. **(E)** Isolated elements (all positive samples).

Placental tissue was sampled according to the criteria proposed by Amsterdam Placental Workshop Group Consensus Statement^17^. Routine histologic sampling included five blocks at least: one block to represent a roll of the extraplacental membranes which are taken from the rupture edge to the placental margin; one block to include three cross-sections of the umbilical cord (one from the fetal end-portion, one from the intermediate portion and one at approximately 3 cm from the placental insertion); three blocks each containing a full-thickness section of normal-appearing placental parenchyma (Fig. 1A–C). All samples were stained with standard hematoxylin and eosin (Fig. 1C).

The diagnosis of acute placental inflammation was based on pathologic criteria. Assessed acute inflammatory lesions of the placenta are defined as follows^2^. Acute funisitis is characterized by infiltration of neutrophils of fetal origin in the umbilical vessel wall (Supplemental Fig. 1), chorioamnionitis is characterized by infiltration of neutrophils of maternal origin in the placental membranes (Supplemental Fig. 2). Chorionic vasculitis is characterized by infiltration of neutrophils of fetal origin in the wall of chorionic vessels (chorionic plate) (Supplemental Fig. 3). The classification system currently in use is that reported by Redline in 2003^18^.

### Microbiological data

Microbiological examinations were performed according to the doctors on duty indications (obstetrician or neonatologist) and always in case of maternal fever, suspected chorioamnionitis, hydrops, or prematurity. If the microbiological examination was requested, samples from the placental disc, membranes, and umbilical cord were cultured. Placentas were sampled in the delivery room, and samples were kept in the appropriate media^19,20^. The hospital’s microbiology laboratory processed all cultures according to standardized protocols using appropriate media for aerobic, anaerobic bacteria, and yeasts^19,20^. Gram staining was performed for all samples. Plates were incubated in dedicated thermostats at 37 °C for 48–72 h. The medium for the anaerobic culture was incubated in a specifically dedicated hood with a controlled atmosphere. Bacterial isolates were identified using Matrix-assisted laser desorption-ionization time-of-flight (MALDI-TOF) instrumentation^19,20^. Chlamydia trachomatis was assessed by conventional molecular assay or multiplex polymerase chain reaction kits^21,22^.

### Clinical management

In our setting, vaginal swabs are usually performed during pregnancy in women with risk factors for preterm labor and delivery. According to the result of the swab, antibiotic therapy can be administered. Premature rupture of membranes and preterm premature rupture of membranes are managed according to international guidelines, and Azithromycin is widely used in premature rupture of membranes and in case of vaginal swab positivity. According to the internal protocol and international guidelines, if an intra-amniotic infection is suspected, the administration of broad-spectrum parenteral antibiotics with coverage for common pathogens is initiated^23,24^. All the included cases were treated according the internal protocols.

### Statistical analysis

All statistical analysis, considering p-value < 0.05 (two-tailed) as statistically significant, were performed with R software (version 3.6.3; R Core Team (2020). R: A language and environment for statistical computing. R Foundation for Statistical Computing, Vienna, Austria. URL https://www.R-project.org/). Continuous variables are presented as the median and interquartile range (IQR) or mean and standard deviation. Dichotomic variables were expressed as frequencies in percentage and absolute values, except those with missing values (NA). Logistic regression results were exhibited as odds ratio (OR) and relative 95% confidence interval (CI.95). Kolmogorov–Smirnov was used to test whether the distribution was parametric or not. The following statistical tests were employed as appropriate: t-test for parametric variables, Wilcoxon test for continuous nonparametric variables, chi-square test, or Fisher exact test. Logistic regression analysis was performed, considering neonatal outcomes as dependent variables and possible risk factors as independent variables. The multivariate model took into account all possible predictive factors with p < 0.05 in univariate analysis (all variables are listed in the multivariate models in Table 4A,B). All variables and their interaction terms were included in the initial multivariate model. When interactions turned out to be nonsignificant, analysis without interaction model was used. Moreover, the false discovery rate test was used to adjust the p-values of the multivariate analysis. Due to the limited number of events in neonatal death, all significant factors were corrected only for gestational age at delivery.

### Ethics approval and consent to participate

The present study was approved by the internal review board of the Department of Medical Area (University of Udine), it was conducted in accordance with Helsinki Declaration and it followed the dictates of the general authorization to process personal data for scientific research purposes by the Italian Data Protection Authority. The need for an informed consent, according with national legislation, was waived by the IRB listed above because this was a retrospective cohort study.

## Results

### Population description

Among the 5910 singleton deliveries in the studied period, the pathological examination was available in 1770 cases. Table 1 reports the characteristics of the population submitted to pathological examination. The mean maternal age at delivery was 33 years (IQR 29–37), and 11.36% of women were > 40 years old. Only 3.39% (60/1770) of these patients exhibited fever (Table 1).

**Table 1:** Clinical, microbiological and histopathologic characteristics.

Maternal age (years)	33 (29–37)
Maternal age > 40 years	11.36% (201/1770)
Nulliparous	60.4% (1069/1770)
**Mode of conception**	
Spontaneous	95.59% (1692/1770)
Ovulation induction /IUI	0.85% (15/1770)
IVF/ICSI	3.56% (63/1770)
** Gestational age at delivery (weeks)**	38 (37–40)
Delivery < 32 weeks	7.51% (133/1770)
Delivery < 34 weeks	12.66% (224/1770)
Delivery < 37 weeks	24.63% (436/1770)
**Macro-regions of origin**	
Italy	71.02% (1257/1770)
East Europa	14.12% (250/1770)
Sub-Saharan Africa	6.27% (111/1770)
Arabian countries	3.05% (54/1770)
Asia	3.62% (64/1770)
Others	1.92% (34/1770)
**Mode of labor**	
Spontaneous	35.5% (628/1769)
Induced	37.54% (664/1769)
Without labor	26.96% (477/1769)
**Mode of delivery**	
Operative vaginal delivery	9.27% (164/1770)
Spontaneous	43.33% (767/1770)
Caesarean section	47.4% (839/1770)
Maternal fever	3.39% (60/1770)
Placental plate microbiology (positive/performed)	27% (98/363)
Placental membranes microbiology (positive/performed)	33.06% (120/363)
Placental cord microbiology (positive/performed)	35.08% (127/362)
**Anatomopathological features**	
Funisitis	10.06% (178/1770)
Chorionic vasculitis (fetal response)	2.71% (48/1770)
**Stage of funisitis**	
Stage 1	63.69% (100/157)
Stage 2	32.48% (51/157)
Stage 3	3.82% (6/157)
**Grade of funisitis**	
Grade 1	85.91% (128/149)
Grade 2	14.09% (21/149)
Acute inflammation of membranes	18.93% (335/1770)
**Chorioamnionitis localization**	
Decidua and chorion	58.51% (196/335)
Amnion and other localizations	41.49% (139/335)
**Stage of chorioamnionitis**	
Stage 1	71.04% (238/335)
Stage 2	24.78% (83/335)
Stage 3	4.18% (14/335)
**Grade of chorioamnionitis**	
Grade 1	75.97% (177/233)
Grade 2	24.03% (56/233)
Funisitis/chorioamnionitis/chorionic vasculitis^a^	20.23% (358/1770)

^a^Considering the total of 5910 pregnancies the prevalence was 6.06% (CI.95 5.48–6.69%).

### Prevalence of acute placental inflammation

Table 1 reports the data concerning histological inflammation. In 178 cases, acute funisitis was diagnosed, which in the population of 5910 pregnancies resulted in 3.01% prevalence (95% CI 2.61–3.48%). Chorionic vasculitis was present in 48 cases with a prevalence of 0.81% (95% CI 0.61–1.08%), and acute inflammation of membranes was present in 335 cases with a prevalence of 5.67% (95% CI 5.11–6.29%). Considering all the 5910 pregnancies, the prevalence of all acute placental inflammations was 6.06% (95% CI 5.48–6.69%). If we consider as denominator only the subgroup of patients in whom histopathological exam was conducted, the prevalence was higher (20.23% 95% CI 18.42–22.16%, 358/1770) (Table 1). Supplemental Tables 1, 2, and 3 show the characteristics of the population according to the presence or absence of funisitis, chorioamnionitis, and chorionic vasculitis. Fever was more prevalent in patients with acute funisitis (15.17% vs. 2.07% in the absence of funisitis), membrane inflammation (8.66% vs. 2.16% in the absence of inflammation), and chorionic vasculitis (16.67% vs. 3.02% in the absence of chorionic vasculitis) than others (Table 2). Acute funisitis was associated with placental membrane inflammation in 89.33% of events and with chorionic vasculitis in 12.36% of events (Table 2). Furthermore, acute funisitis was associated with severe chorioamnionitis (high stage and grade, Table 2). Besides, both stage and degree of inflammation of membranes were higher in the presence than in the absence of chorionic vasculitis (Table 2).

**Table 2:** Clinical, microbiological and histopathologic characteristics divided by the presence or absence of: (A) funisitis, (B) chorioamnionitis, (C) chorionic vasculitis.

(A)	Funisitis absent (1592)	Funisitis present (178)	p
Maternal fever	2.07% (33/1592)	15.17% (27/178)	< 0.05
Placental plate microbiology (positive/performed)	23.02% (67/291)	43.06% (31/72)	< 0.05
Placental membranes microbiology (positive/performed)	28.97% (84/290)	49.32% (36/73)	< 0.05
Placental cord microbiology (positive/performed)	31.38% (91/290)	50.00% (36/72)	< 0.05
**Anatomopathological features**			
Chorionic vasculitis	1.63% (26/1592)	12.36% (22/178)	< 0.05
Chorioamnionitis	11.06% (176/1592)	89.33% (159/178)	< 0.05
**Chorioamnionitis localization**			
Capsular decidua and chorion	77.27% (136/176)	37.74% (60/159)	< 0.05
Amnion and other locations	22.73% (40/176)	62.26% (99/159)	< 0.05
**Chorioamnionitis stage and grade**			
Stage 1	84.66% (149/176)	55.97% (89/159)	< 0.05
Stage 2	14.20% (25/176)	36.48% (58/159)	< 0.05
Stage 3	1.14% (2/176)	7.55% (12/159)	< 0.05
Grade 1	88.50% (100/113)	64.17% (77/120)	< 0.05
Grade 2	11.50% (13/113)	35.83% (43/120)	< 0.05

(B)	Chorioamnionitis absent (1435)	Chorioamnionitis present (335)	p
Maternal fever	2.16% (31/1435)	8.66% (29/335)	< 0.05
Placental plate microbiology (positive/performed)	20.97% (52/248)	40.00% (46/115)	< 0.05
Placental membranes microbiology (positive/performed)	28.23% (70/248)	43.48% (50/115)	< 0.05
Placental cord microbiology (positive/performed)	30.36% (75/247)	45.22% (52/115)	< 0.05
**Anatomopathological features**			
Funisitis	1.32% (19/1435)	47.46% (159/335)	< 0.05
Chorionic vasculitis (fetal response)	0.35% (5/1435)	12.84% (43/335)	< 0.05
**Funisitis stage and grade**			
Stage 1	64.29% (9/14)	63.64% (91/143)	0.962
Stage 2	35.71% (5/14)	32.17% (46/143)	0.787
Stage 3	0.00% (0/14)	4.20% (6/143)	0.435
Grade 1	100.00% (13/13)	84.56% (115/136)	0.126
Grade 2	0.00% (0/13)	15.44% (21/136)	0.126

(C)	Chorionic vasculitis absent (1722)	Chorionic vasculitis present (48)	p
Maternal fever	3.02% (52/1722)	16.67% (8/48)	< 0.05
Placental plate microbiology (positive/performed)	25.95% (89/343)	45.00% (9/20)	0.062
Placental membranes microbiology (positive/performed)	31.78% (109/343)	55.00% (11/20)	< 0.05
Placental cord microbiology (positive/performed)	34.50% (118/342)	45.00% (9/20)	0.339
**Anatomopathological features**			
Funisitis	9.06% (156/1722)	45.83% (22/48)	< 0.05
Chorioamnionitis	16.96% (292/1722)	89.58% (43/48)	< 0.05
**Chorioamnionitis localization**			
Decidua and chorion	59.59% (174/292)	51.16% (22/43)	0.295
Amnion and other localizations	40.41% (118/292)	48.84% (21/43)	0.295
**Chorioamnionitis stage and grade**			
Stage 1	73.63% (215/292)	53.49% (23/43)	< 0.05
Stage 2	23.63% (69/292)	32.56% (14/43)	0.205
Stage 3	2.74% (8/292)	13.95% (6/43)	< 0.05
Grade 1	78.46% (153/195)	63.16% (24/38)	< 0.05
Grade 2	21.54% (42/195)	36.84% (14/38)	< 0.05

**Table 3:** Pregnancy outcome divided by the presence or absence of: (A) funisitis, (B) chorioamnionitis, (C) chorionic vasculitis.

(A)	Funisitis absent (1592)	Funisitis present (178)	p
Male newborn	52.76% (840/1592)	50.56% (90/178)	0.577
1 min Apgar	8.00 (7.00–9.00)	7.00 (5.00–9.00)	< 0.05
5 min Apgar	9.00 (8.00–9.00)	8.00 (7.00–9.00)	< 0.05
Neonatal weight (g)	2940.89 (± 770.57)	2792.73 (± 1057.91)	0.071
Neonatal weight (MoM)	0.98 (± 0.15)	1.01 (± 0.15)	< 0.05
Placental weight (g)	523.95 (± 159.03)	526.49 (± 178.19)	0.888
SGA < 3rd percentile	5.60% (89/1589)	1.69% (3/178)	< 0.05
SGA < 10th percentile	16.87% (268/1589)	12.92% (23/178)	0.178
LGA > 90th percentile	10.64% (169/1589)	12.92% (23/178)	0.353
LGA > 97th percentile	4.91% (78/1589)	6.74% (12/178)	0.292
IUGR	7.29% (116/1591)	2.25% (4/178)	< 0.05
Neonatal cardiopulmonary resuscitation	13.20% (210/1591)	31.46% (56/178)	< 0.05
NICU admission	19.41% (309/1592)	35.39% (63/178)	< 0.05
**Neonatal outcome**			
Alive	98.55% (1566/1589)	94.92% (168/177)	< 0.05
Stillbirth	0.69% (11/1589)	1.13% (2/177)	0.518
Postnatal death	0.76% (12/1589)	3.95% (7/177)	< 0.05

(B)	Chorioamnionitis absent (1435)	Chorioamnionitis present (335)	p
Male newborn	52.06% (747/1435)	54.63% (183/335)	0.396
1 min Apgar	8.00 (7.00–9.00)	8.00 (6.00–9.00)	< 0.05
5 min Apgar	9.00 (8.00–9.00)	9.00 (8.00–9.00)	< 0.05
Neonatal weight (g)	2949.45 (± 741.84)	2825.50 (± 1027.99)	< 0.05
Neonatal weight (MoM)	0.98 (± 0.15)	1.00 (± 0.16)	< 0.05
Placental weight (g)	529.48 (± 157.44)	500.58 (± 175.41)	< 0.05
SGA < 3rd percentile	5.38% (77/1432)	4.48% (15/335)	0.505
SGA < 10th percentile	16.55% (237/1432)	16.12% (54/335)	0.848
LGA > 90th percentile	10.13% (145/1432)	14.03% (47/335)	< 0.05
LGA > 97th percentile	4.61% (66/1432)	7.16% (24/335)	0.055
IUGR	6.97% (100/1434)	5.97% (20/335)	0.511
Neonatal cardiopulmonary resuscitation	12.06% (173/1434)	27.76% (93/335)	< 0.05
NICU admission	19.09% (274/1435)	29.25% (98/335)	< 0.05
**Neonatal outcome**			
Alive	98.74% (1414/1432)	95.81% (320/334)	< 0.05
Stillbirth	0.63% (9/1432)	1.20% (4/334)	0.273
Postnatal death	0.63% (9/1432)	2.99% (10/334)	< 0.05

(C)	Chorionic vasculitis absent(1722)	Chorionic vasculitis present (48)	p
Male newborn	52.67% (907/1722)	47.92% (23/48)	0.515
1 min Apgar	8.00 (7.00–9.00)	7.50 (5.00–8.00)	< 0.05
5 min Apgar	9.00 (8.00–9.00)	9.00 (8.00–9.00)	0.064
Neonatal weight (g)	2928.65 (± 794.87)	2830.73 (± 1115.75)	0.549
Neonatal weight (MoM)	0.98 (± 0.15)	1.03 (± 0.15)	< 0.05
Placental weight (g)	525.41 (± 159.85)	483.32 (± 200.26)	0.280
SGA < 3rd percentile	5.35% (92/1719)	0.00% (0/48)	0.100
SGA < 10thpercentile	16.70% (287/1719)	8.33% (4/48)	0.123
LGA > 90th percentile	10.59% (182/1719)	20.83% (10/48)	< 0.05
LGA > 97th percentile	4.89% (84/1719)	12.50% (6/48)	< 0.05
IUGR	6.97% (120/1721)	0.00% (0/48)	0.058
Neonatal cardiopulmonary resuscitation	14.70% (253/1721)	27.08% (13/48)	< 0.05
NICU admission	20.91% (360/1722)	25.00% (12/48)	0.492
**Neonatal outcome**			
Alive	98.43% (1691/1718)	89.58% (43/48)	< 0.05
Stillbirth	0.70% (12/1718)	2.08% (1/48)	0.268
Postnatal death	0.87% (15/1718)	8.33% (4/48)	< 0.05

### Association between acute placental inflammation and microbiological positivity

The microbiological examination was done in 363 cases (20.51%) for both placental disc and membranes and in 362 cases (20.45%) for the umbilical cord. The microbiological examination was positive in 27.00% of cases for the placental disc, 33.06% for membranes, and in 35.08% for the umbilical cord (Table 1). Among the 358 cases of acute placental inflammation on histological examination, a microbiological examination was conducted in 125 cases, and it resulted positive in 64 cases. More specifically, the placental disc swab was positive in 39.02% (48/123) of the cases (95% CI 30.86–47.85%), the membrane swab was found positive in 43.55% (54/124) of the cases (95% CI 35.15–52.34%), and the umbilical cord swab was positive in 44.72% (55/123) of the cases (95% CI 36.22–53.53%) (Fig. 1D). In total, 15 pathogenic microbial genera were identified, including the genus *Candida* and 14 genera of bacteria. In 50 cases, the infections were monomicrobial and polymicrobial in 14 cases. In Fig. 1E, the prevalence of pathogenic genera is shown considering all the samples. *Escherichia* was the most common genus, with a prevalence of 39.06% of the total samples (all were *Escherichia coli*). The second in frequency was the genus *Streptococcus*, with a total frequency of 31.25%; in particular, among the different species, the *Streptococcus agalactiae* (*group B streptococcus* or GBS) was predominant, with a frequency of 17%. The microbiological examination was positive in 31.38% of cases without acute funisitis and in 17.28% of cases without chorioamnionitis.

### Acute placental inflammation and maternal–fetal outcomes

Table 3 shows pregnancy outcomes according to the presence or absence of funisitis, chorioamnionitis, and chorionic vasculitis. In the presence of acute inflammation of placental tissues, there was a greater need for neonatal cardiopulmonary resuscitation, admission to neonatal intensive care unit, and postnatal death of the newborn. Table 4 displays bivariate and multivariate analysis results conducted by logistic regression of the following outcomes, considered dependent variables: neonatal cardiopulmonary resuscitation, neonatal intensive care unit admission, and postnatal death of the newborn. Acute inflammation of membranes was an independent risk factor for neonatal cardiopulmonary resuscitation (OR 2.12; CI.95 1.36–3.31); furthermore, high grading was at increased risk of neonatal cardiopulmonary resuscitation (grade 2 vs. grade 1 OR 4.96; CI.95 2.59–9.53; p < 0.05). Acute funisitis was an independent risk factor for admission to intensive neonatal care unit (OR 3.2; CI.95 1.67–6.12), besides high grading was at increased risk of intensive neonatal care unit hospitalization (grade 2 vs. grade 1 OR 2.88; CI.95 1.07–7.75; p < 0.05). Chorionic vasculitis was an independent risk factor for postnatal death of the newborn, after controlling for gestational age at birth (OR 5.38; CI.95 1.37–21.06); moreover, high grading was at increased risk of postnatal death of the newborn (grade 2 vs. grade 1 OR 11.62; CI.95 1.8–74.93; p < 0.05).

**Table 4:** Univariate and multivariate logistic regression analysis.

(A)	OR (CI.95)	p	OR (CI.95) (†)	p (†)	p-adj (‡)
Maternal age(years)	1 (0.97–1.02)	0.792			
Nulliparous	1.45 (1.1–1.91)	< 0.05	1.67 (1.21–2.33)	< 0.05	< 0.05
Origin: Sub-Saharan Africa and others category	1.79 (1.14–2.81)	< 0.05	1.44 (0.83–2.49)	0.190	0.285
Induced labor/augmentation	0.45 (0.34–0.62)	< 0.05	0.92 (0.64–1.32)	0.650	0.730
Cesarean section	2.41 (1.83–3.17)	< 0.05	1.93 (1.39–2.66)	< 0.05	< 0.05
PRHD	0.45 (0.29–0.68)	< 0.05	0.99 (0.59–1.68)	0.980	0.978
Male newborn	1.08 (0.83–1.41)	0.546			
Weight at birth (MoM)	0.74 (0.32–1.74)	0.496			
**Mode of conception**					
Spontaneous	Reference				
Induction ovulation/IUI	0.88 (0.2–3.93)	0.867			
IVF/ICSI	1.49 (0.8–2.78)	0.213			
IUGR	1.47 (0.92–2.34)	0.110			
Gestational age at delivery (weeks)	0.76 (0.73–0.79)	< 0.05	0.77 (0.74–0.81)	< 0.05	< 0.05
Acute funisitis	3 (2.11–4.26)	< 0.05	1.62 (0.95–2.77)	0.070	0.135
Chorionic vasculitis	2.21 (1.15–4.26)	< 0.05	0.73 (0.3–1.76)	0.480	0.623
Acute inflammation of membranes	2.81 (2.1–3.75)	< 0.05	2.12 (1.36–3.31)	< 0.05	< 0.05

(B)	OR (CI.95)	p	OR (CI.95) (†)	p (†)	p-adj (‡)
Maternal age (years)	0.98 (0.96–1.01)	0.146			
Nulliparous	1.26 (0.99–1.6)	0.061			
Origin: Sub-Saharan Africa and others category	1.27 (0.82–1.97)	0.281			
Induced labor/augmentation	0.3 (0.23–0.4)	< 0.05	0.75 (0.52–1.09)	0.140	0.189
Cesarean section	2.25 (1.78–2.85)	< 0.05	1.43 (1.03–1.98)	< 0.05	0.053
PRHD	0.31 (0.21–0.46)	< 0.05	0.93 (0.53–1.62)	0.780	0.916
Male newborn	0.91 (0.73–1.15)	0.435			
Weight at birth (MoM)	0.73 (0.35–1.54)	0.411			
**Mode of conception**					
Spontaneous	Reference				
Ovulation induction /IUI	1.39 (0.44–4.4)	0.573			
IVF/ICSI	1.53 (0.88–2.68)	0.136			
IUGR	2.01 (1.35–3)	< 0.05	2.46 (1.46–4.16)	< 0.05	< 0.05
Gestational age at delivery (weeks)	0.58 (0.55–0.62)	< 0.05	0.59 (0.55–0.63)	< 0.05	< 0.05
Acute funisitis	2.27 (1.63–3.17)	< 0.05	3.2 (1.67–6.12)	< 0.05	< 0.05
Chorionic vasculitis	1.3 (0.67–2.52)	0.446			
Acute inflammation of membranes	1.76 (1.34–2.31)	< 0.05	1.02 (0.6–1.72)	0.950	0.950

(C)	OR (CI.95)	p	OR (CI.95) (†)	p (†)	p-adj (‡)
Gestational age at delivery (weeks)	0.74 (0.68–0.81)	< 0.05	0.76 (0.70–0.84) (*)	< 0.05	–
All acute inflammations	4.51 (1.82–11.17)	< 0.05	2.57 (0.88–7.54) (**)	0.086	–
Acute funisitis	5.44 (2.11–14)	< 0.05	2.81 (0.94–8.4) (**)	0.065	–
Chorionic vasculitis	10.49 (3.34–32.91)	< 0.05	5.38 (1.37–21.06) (**)	< 0.05	–
Acute inflammation of membranes	4.91 (1.98–12.18)	< 0.05	2.73 (0.93–8.06) (**)	0.070	–

(A) Risk factor for neonatal cardiopulmonary resuscitation. (B) Risk factors for NICU hospitalization. (C) Risk factors for post-natal death. The data are reported as odds ratio (OR) and the relative 95% confidence interval (CI.95). This table shows univariate logistic regression analysis and multivariate logistic regression analysis (†). Furthermore, the multivariate logistic regression analysis p-values are adjusted using the false discovery rate test (‡).

(*) Corrected for all acute inflammations; (**) Corrected for gestational age at birth.

## Discussion

The prevalence of acute placental inflammation was 6.06% considering all singleton deliveries in the study time frame and 20.23% considering only examined placentas. Microbiological analyses were negative in about half of examined placentas. Our multivariate analysis confirmed the association between inflammation and adverse neonatal outcome. Controlling for multiple confounders, acute inflammation of membranes was an independent risk factor for neonatal cardiopulmonary resuscitation (OR 2.12; CI.95 1.36–3.31; p < 0.05), acute funisitis was an independent risk factor for admission to intensive neonatal care unit (OR 3.2; CI.95 1.67–6.12; p < 0.05), and chorionic vasculitis was an independent risk factor for postnatal death of the newborn (OR 5.38; CI.95 1.37–21.06; p < 0.05).

The main limitation of this study is its retrospective design, which could have led to information bias. Sampling placental tissues for microbiological cultures differs from many studies in literature, which used amniotic fluid cultures, limiting the possibility of directly comparing these results with the literature data. This approach, though, gave us the possibility to analyze a vast amount of data retrospectively. Our research’s strong points are the relatively large sample size and the homogeneous assessment of the samples, which was ensured through a strict clinical protocol. Moreover, through multivariate statistical analysis, we were able to control the effect of multiple confounding factors.

The prevalence rate of acute placental inflammation was 20% similar to the findings of other authors 26–27%^25,26^. However, this study’s prevalence is lower probably because we considered all placentas and not just those sent for clinically suspected chorioamnionitis. Meanwhile, the prevalence of acute inflammation histopathological finding overall is lower and accounts for 6.06% of all singleton deliveries. This result differs only slightly from the estimates of Woodd et al., who reported an incidence of 3.9%, and from the estimates of Russel et al., who examined 7505 placentas from singleton deliveries after 20 weeks of gestation and reported chorioamnionitis in 5.2% of all cases^2,27,28^. Among the 125 cases of acute placental inflammation that underwent microbiological examination, 51.2% resulted positive. This percentage appears relatively high in respect to the previous literature but must be considered in the context of our retrospective setting: in fact, the placental samples were sent for microbiological examination only in case of suspected infection, unlike to prospective studies^25^. Acute placental inflammations have negative microbiological results in about half of cases; these cases’ etiology is yet to be clarified. First of all, acute chorioamnionitis can occur as a sterile intraamniotic inflammation induced by signals released under cellular stress, injury, or death. Secondly, the use of antibiotic therapies may affect the viability of bacteria with consequent negative microbiological results. Lastly, atypical pathogens such as Mycoplasma or Ureaplasma spp. do not grow if not in particular culture media^2,25,29–31^. Conventional cultivation techniques cannot identify the entire microbial diversity of an ecological niche because the conditions required for the growth of microorganisms in vitro are unknown, and some organisms are, therefore, considered non-culturable^32,33^. A possible role of these microorganisms present below the level of detection of culture and 16S PCR, and that can be highlighted only through deep DNA and RNA sequencing, remains to be addressed. Conversely, the cases of positive bacteriology with negative histologic analysis (31.38% for the umbilical cord and 28.23% for membranes examination) could be explained by sample contamination, tissue contiguity, or an early stage of infection where pathogens have not yet caused visible lesions^25^.

We found that *Escherichia* and *Streptococcus* were the most common genera; in particular, *group B streptococcus* was predominant among the different *Streptococcus species*. This finding is consistent with some studies in literature but in contrast with others. Bhola et al. and Sperling et al. reported *Group B Streptococcus* and *Escherichia Coli* as the most frequently identified bacteria. Conversely, Romero et al. found that *Ureaplasma spp.* and *Gardnerella vaginalis* are the most commonly identified microorganisms by cultivation and PCR/ESI–MS, respectively. According to other studies, *Ureaplasma spp.* and *Mycoplasma spp.* are the most common microorganisms. In our reality, the use of prophylaxis or therapy with Azithromycin may have affected these bacteria’s viability with consequent negative microbiological results^4,26,30,34–37^.

The significant prevalence of fever in patients with acute placental inflammation in our sample is also widely reported in the literature, and this symptom is the cornerstone in the clinical diagnosis of chorioamnionitis^3,4,8^. Vice versa, isolated maternal fever is not necessarily the index of chorioamnionitis, and indeed it can lead to wrong diagnoses and subsequent overtreatment, which become particularly relevant in preterm gestations^1^.

In our study, fetal responses, represented by funisitis and chorionic vasculitis, relate with a higher stage and degree of membrane inflammation on the maternal counterpart. This is widely reported in literature^38,39^. Other published data demonstrate the association between chorionic vasculitis and stillbirth, as we also found, despite the lack of statistical significance. However, the link with postnatal mortality that we found is little present in literature^40^. Lau et al. reported that neonatal mortality was more significant when fetal inflammation was present (chorionic funisitis and vasculitis considered together) compared to maternal inflammation alone, with a postnatal death prevalence of 9.2% in cases with fetal inflammation, vs. 7.2% in cases with maternal inflammation (vs. 3.6% in the absence of acute placental inflammation)^39^. In our study, correcting for the gestational age, only the chorionic plate’s vasculitis was a risk factor for neonatal death. This finding could be related to the ascending pathway of infection so that the placenta’s histologic inflammation would be the last step of the progressing pathogenic noxa. The etiopathogenesis that links placental vasculitis with postnatal mortality is not clear yet. Some authors hypothesized that an increasing duration or severity of placental histopathology compatible with fetal inflammatory response syndrome (FIRS) could have a dose-dependent relationship with death in preterm infants^38^. FIRS is a condition characterized by systemic activation of the fetal innate immune system^41^. In other terms it is the fetal counterpart of systemic inflammatory response syndrome^41^. FIRS can lead to multiple organ dysfunction, including adrenal glands, heart, brain, lungs, and skin, and it correlates with higher neonatal morbidity, and mortality^39,41,42^. Initially, chorioamnionitis shows a maternal inflammatory response. In more severe cases, fetal microbial invasion can result in a FIRS, which originates from the placenta’s fetal vascular component, initially from the umbilical vein, then the umbilical arteries and vessels of the chorionic plate. As regard to neonatal outcomes, many studies focused on the acute inflammation of placental tissues, finding an association between chorioamnionitis and neonatal morbidity in terms of pneumonia, brain injury, or sepsis. Thus, chorioamnionitis leads to a greater need for neonatal cardiopulmonary resuscitation and admission to neonatal intensive care unit, in line with our results^1,41,43–46^.

Our data advise that placental inflammation (whether associated with infectious or non-infectious origins) could lead to placental dysfunction^47^. This placental dysfunction can be the reason for the adverse outcomes associated with placental inflammation. Therefore, our data suggest two possible clinical research purposes. First, improving our etiological knowledge on placental inflammation of non-infectious origin. Hence understanding the etiology of non-infectious acute placental inflammation, which according to our results, occurs in half of the cases, appears to be the keystone on which future prospective studies in this field should focus. Second, therapeutic strategies that act at reducing placental inflammation, as previously suggested, could also improve the negative outcomes assessed in this study^47^.

## Conclusions

Chorioamnionitis is a common and not minor complication of pregnancy with a prevalence in this study of 6.06%. It is associated with newborn adverse outcomes and postnatal death, regardless of whether it originates from infection or sterile inflammation. Chorionic vasculitis is independently associated with postnatal death. This finding, so far poorly explored, seems crucial and raises new questions on the possible role of an exaggerated and progressive fetal response, of which chorionic vasculitis could be the histological display, in determining the infant’s outcome.

## Supplementary Information

Supplementary Figure S1.

Supplementary Figure S2.

Supplementary Figure S3.

Supplementary Legends.

Supplementary Tables.

## Data Availability

The data that support the findings of this study are available, but restrictions apply to the availability of these data, which was used under license for the current study, and so are not publicly available. Data are however available from the authors upon reasonable request and with permission of the Internal Review Board.

## Abbreviations

CI: Confidence interval
CI.95: 95% confidence interval
FIRS: Fetal inflammatory response syndrome
GBS: Group B streptococcus
ICSI: Intracytoplasmic sperm injection
IQR: Interquartile range
IUFD: Intrauterine fetal demise
IUGR: Intrauterine growth restriction
IUI: Intrauterine insemination
IVF: In vitro fertilization
LGA: Large for gestational age
MoM: Multiple of the median
NA: Missing values
NICU: Neonatal intensive care unit
OR: Odds ratio
PRHDs: Pregnancy-related hypertensive disorders
SGA: Small for gestational age

## References

[1] Higgins RD, Saade G, Polin RA, Grobman WA, Buhimschi IA, Watterberg K (2016). Evaluation and management of women and newborns with a maternal diagnosis of chorioamnionitis: Summary of a workshop. Obstet. Gynecol..

[2] Kim CJ, Romero R, Chaemsaithong P, Chaiyasit N, Yoon BH, Kim YM (2015). Acute chorioamnionitis and funisitis: Definition, pathologic features, and clinical significance. Am. J. Obstet. Gynecol..

[3] Gibbs RS, Duff P (1991). Progress in pathogenesis and management of clinical intraamniotic infection. Am. J. Obstet. Gynecol..

[4] Tita ATN, Andrews WW (2010). Diagnosis and management of clinical chorioamnionitis. Clin. Perinatol..

[5] Romero R, Miranda J, Chaiworapongsa T, Chaemsaithong P, Gotsch F, Dong Z (2014). A novel molecular microbiologic technique for the rapid diagnosis of microbial invasion of the amniotic cavity and intra-amniotic infection in preterm labor with intact membranes. Am. J. Reprod. Immunol..

[6] Romero R, Miranda J, Chaemsaithong P, Chaiworapongsa T, Kusanovic JP, Dong Z (2015). Sterile and microbial-associated intra-amniotic inflammation in preterm prelabor rupture of membranes. J. Matern. Fetal Neonatal Med..

[7] Soper DE, Mayhall CG, Dalton HP (1989). Risk factors for intraamniotic infection: A prospective epidemiologic study. Am. J. Obstet. Gynecol..

[8] Newton ER (1993). Chorioamnionitis and intraamniotic infection. Clin. Obstet. Gynecol..

[9] Fahey JO (2008). Clinical management of intra-amniotic infection and chorioamnionitis: A review of the literature. J. Midwifery Women’s Health..

[10] Londero AP, Rossetti E, Pittini C, Cagnacci A, Driul L (2019). Maternal age and the risk of adverse pregnancy outcomes: A retrospective cohort study. BMC Pregnancy Childbirth..

[11] Londero AP, Bertozzi S, Visentin S, Fruscalzo A, Driul L, Marchesoni D (2013). High placental index and poor pregnancy outcomes: A retrospective study of 18,386 pregnancies. Gynecol. Endocrinol..

[12] The American College of Obstetricians and Gynecologists (2020). Gestational hypertension and preeclampsia: ACOG practice bulletin summary, number 222. Obstet. Gynecol..

[13] Bertozzi S, Londero AP, Salvador S, Grassi T, Fruscalzo A, Driul L (2011). Influence of the couple on hypertensive disorders during pregnancy: A retrospective cohort study. Pregnancy Hypertens. Int. J. Women’s Cardiovasc. Health..

[14] Visentin S, Londero AP, Calanducci M, Grisan E, Bongiorno MC, Marin L (2019). Fetal abdominal aorta: Doppler and structural evaluation of endothelial function in intrauterine growth restriction and controls. Ultraschall Med..

[15] Barfield WD, Newborn CoFA. Standard terminology for fetal, infant, and perinatal deaths. *Pediatrics* 137 (2016). 10.1542/peds.2016-0551 27244834

[16] Baergen RN (2011). Manual of Pathology of the Human Placenta.

[17] Khong TY, Mooney EE, Ariel I, Balmus NCM, Boyd TK, Brundler MA (2016). Sampling and definitions of placental lesions: Amsterdam Placental Workshop Group consensus statement. Arch. Pathol. Lab. Med..

[18] Redline RW, Faye-Petersen O, Heller D, Qureshi F, Savell V, Vogler C (2003). Amniotic infection syndrome: Nosology and reproducibility of placental reaction patterns. Pediatr. Dev. Pathol..

[19] Cornaglia G, Courcol R, Hermann JL, Kahlmeter G, Peigue-Lafeuille H, Vila J (2012). European Manual of Clinical Microbiology.

[20] Leber, A.L. *Clinical Microbiology Procedures Handbook*. (2016).

[21] Meyer T, Buder S (2020). The laboratory diagnosis of neisseria gonorrhoeae: Current testing and future demands. Pathogens..

[22] Screm, M., Santolo, M.D., Scarparo, C., Arzese, A. P2.047 evaluation of a multiplex real-time PCR assay for rapid detection of *C. trachomatis* and *N. gonorrhoeae* from genital clinical specimens. *Sex Transm. Infect*. **89**, A102.2–A102 (2013).

[23] The American College of Obstetricians and Gynecologists (2020). Prelabor rupture of membranes: ACOG practice bulletin summary, number 217. Obstet. Gynecol..

[24] The American College of Obstetricians and Gynecologists. Committee opinion no. 712: Intrapartum management of intraamniotic infection. *Obstet Gynecol.* **130**, e95–e101 (2017). 10.1097/AOG.0000000000002236 28742677

[25] da Mota VQ, Prodhom G, Yan P, Hohlfheld P, Greub G, Rouleau C (2013). Correlation between placental bacterial culture results and histological chorioamnionitis: A prospective study on 376 placentas. J. Clin. Pathol..

[26] Bhola K, Al-Kindi H, Fadia M, Kent AL, Collignon P, Dahlstrom JE (2008). Placental cultures in the era of peripartum antibiotic use. Aust. N. Z. J. Obstet. Gynaecol..

[27] Russel P (1979). Inflammatory lesions of the human placenta. I. Clinical significance of acute chorioamnionitis. Am. J. Diagn. Gynecol. Obstet..

[28] Woodd S.L. *et al*. Incidence of maternal peripartum infection: A systematic review and meta-analysis. *PLoS Med*. **2019**, 16 (2019). 10.1371/journal.pmed.1002984 PMC6903710 31821329

[29] Romero R, Salafia CM, Athanassiadis AP, Hanaoka S, Mazor M, Sepulveda W (1992). The relationship between acute inflammatory lesions of the preterm placenta and amniotic fluid microbiology. Am. J. Obstet. Gynecol..

[30] Romero R, Miranda J, Chaiworapongsa T, Chaemsaithong P, Gotsch F, Dong Z (2015). Sterile intra-amniotic inflammation in asymptomatic patients with a sonographic short cervix: Prevalence and clinical significance. J. Matern. Fetal Neonatal Med..

[31] Romero R, Miranda J, Kusanovic JP, Chaiworapongsa T, Chaemsaithong P, Martinez A (2015). Clinical chorioamnionitis at term I: Microbiology of the amniotic cavity using cultivation and molecular techniques. J. Perinat. Med..

[32] Stewart EJ (2012). Growing unculturable bacteria. J. Bacteriol..

[33] Romero R, Gomez-Lopez N, Kusanovic JP, Pacora P, Panaitescu B, Erez O (2018). Clinical chorioamnionitis at term: New insights into the etiology, microbiology, and the fetal, maternal and amniotic cavity inflammatory responses. Nogyogyaszati Szuleszeti Tovabbkepzo Szemle..

[34] Oh KJ, Kim SM, Hong JS, Maymon E, Erez O, Panaitescu B (2017). Twenty-four percent of patients with clinical chorioamnionitis in preterm gestations have no evidence of either culture-proven intraamniotic infection or intraamniotic inflammation. Am. J. Obstet. Gynecol..

[35] Schubert, P.T. Spectrum of changes seen with placental intravascular organisms. *Pediatr. Dev. Pathol*. **22**, 229–235 (2019). 10.1177/1093526618801616 30334666

[36] Waites KB, Katz B, Schelonka RL (2005). Mycoplasmas and ureaplasmas as neonatal pathogens. Clin. Microbiol. Rev..

[37] Sperling RS, Newton E, Gibbs RS (1988). Intraamniotic infection in low-birth-weight infants. J. Infect. Dis..

[38] Salas AA, Faye-Petersen OM, Sims B, Peralta-Carcelen M, Reilly SD, McGwin G (2013). Histologic characteristics of the fetal inflammatory response associated with neurodevelopmental impairment and death in extremely preterm infants. J. Pediatr..

[39] Lau J, Magee F, Qiu Z, Houbé J, Dadelszen PV, Lee SK (2005). Chorioamnionitis with a fetal inflammatory response is associated with higher neonatal mortality, morbidity, and resource use than chorioamnionitis displaying a maternal inflammatory response only. Am. J. Obstet. Gynecol..

[40] Moyo SR, Hägerstrand I, Nyström L, Tswana SA, Blomberg J, Bergström S (1996). Stillbirths and intrauterine infection, histologic chorioamnionitis and microbiological findings. Int. J. Gynecol. Obstet..

[41] Gotsch F, Romero R, Kusanovic JP, Mazaki-Tovi S, Pineles BL, Erez O (2007). The fetal inflammatory response syndrome. Clin. Obstet. Gynecol..

[42] Ducey J, Owen A, Coombs R, Cohen M (2015). Vasculitis as part of the fetal response to acute chorioamnionitis likely plays a role in the development of necrotizing enterocolitis and spontaneous intestinal perforation in premature neonates. Eur. J. Pediatr. Surg..

[43] Yoder PR, Gibbs RS, Blanco JD, Castaneda YS, Clair PJS (1983). A prospective, controlled study of maternal and perinatal outcome after intra-amniotic infection at term. Am. J. Obstet. Gynecol..

[44] Morales WJ, Washington SR, Lazar AJ (1987). The effect of chorioamnionitis on perinatal outcome in preterm gestation. J. Perinatol..

[45] Alexander JM, McIntire DM, Leveno KJ (1999). Chorioamnionitis and the prognosis for term infants. Obstet. Gynecol..

[46] Raines DA, Wagner A, Salinas A (2017). Intraamniotic infection and the term neonate. Neonatal Netw..

[47] Sibley CP (2017). Treating the dysfunctional placenta. J. Endocrinol..

