# Supplementary figures and images for: Placental acute inflammation infiltrates and pregnancy outcomes: a retrospective cohort study
Source: Sci Rep. 2021 Dec 17;11:24165. doi: 10.1038/s41598-021-03655-4 (PMC8683439; doi:10.1038/s41598-021-03655-4)

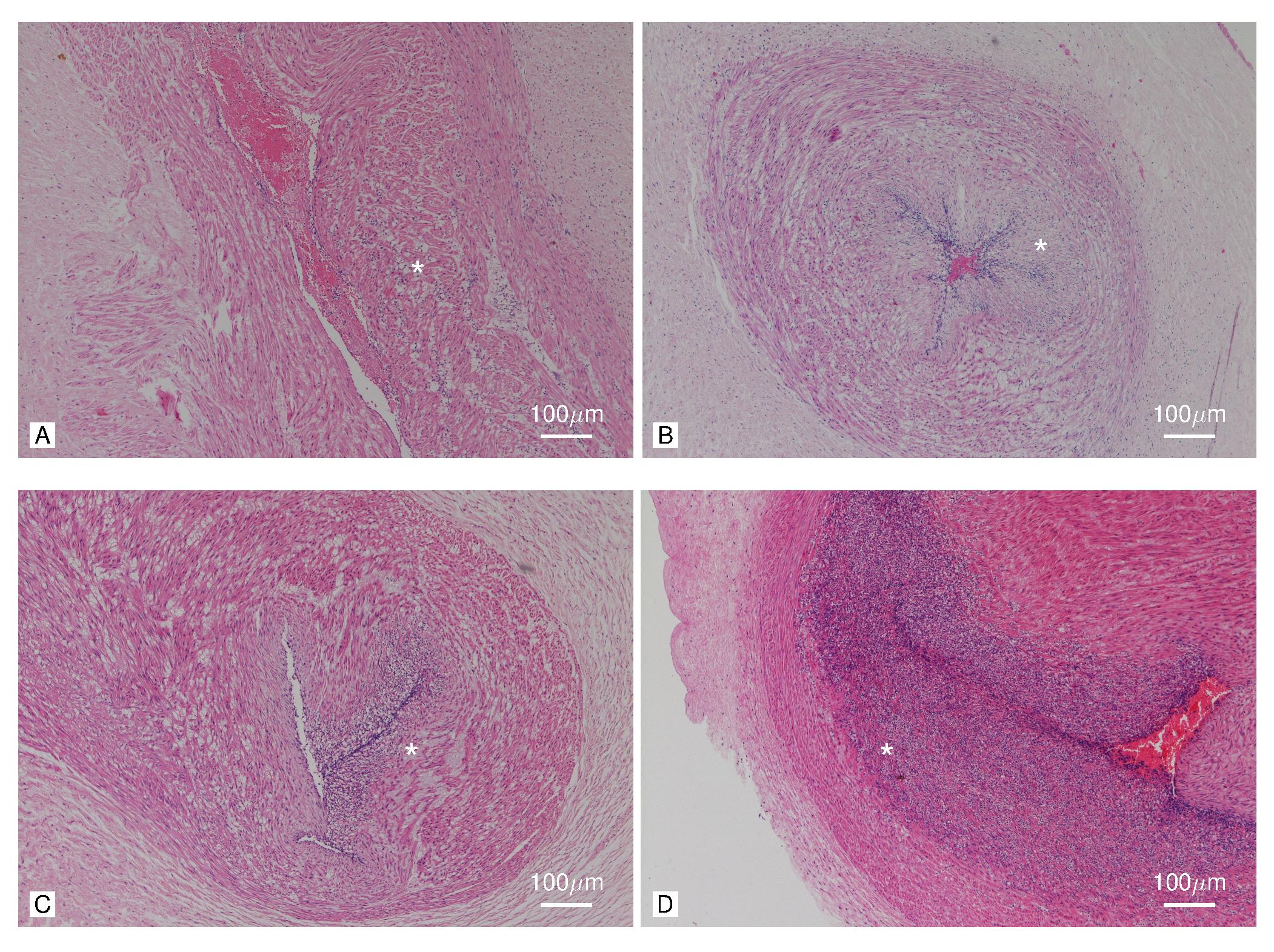

Supplement: Supplementary file 1 — Supplementary Figure S1. [file 41598_2021_3655_MOESM1_ESM.jpg]

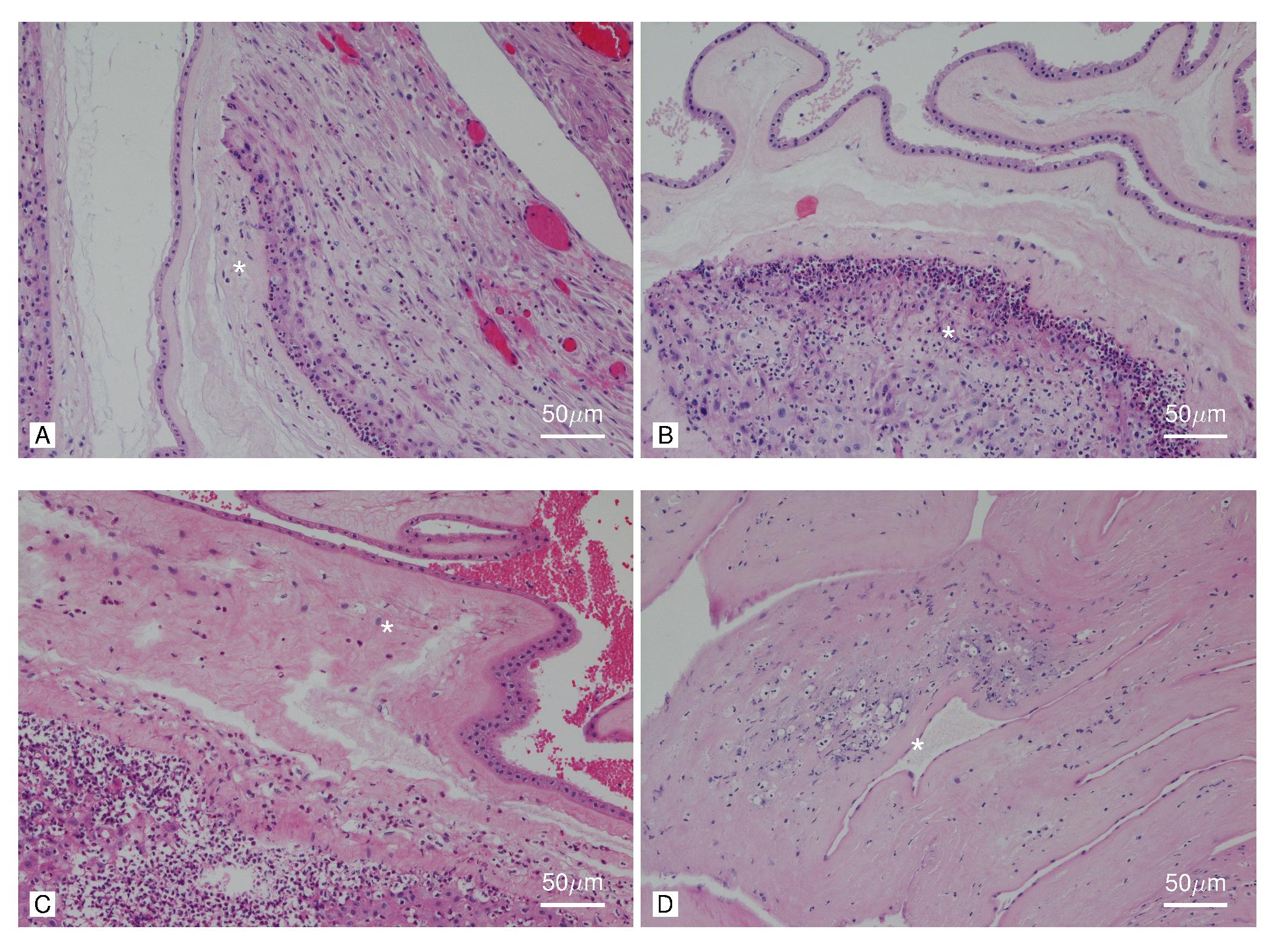

Supplement: Supplementary file 2 — Supplementary Figure S2. [file 41598_2021_3655_MOESM2_ESM.jpg]

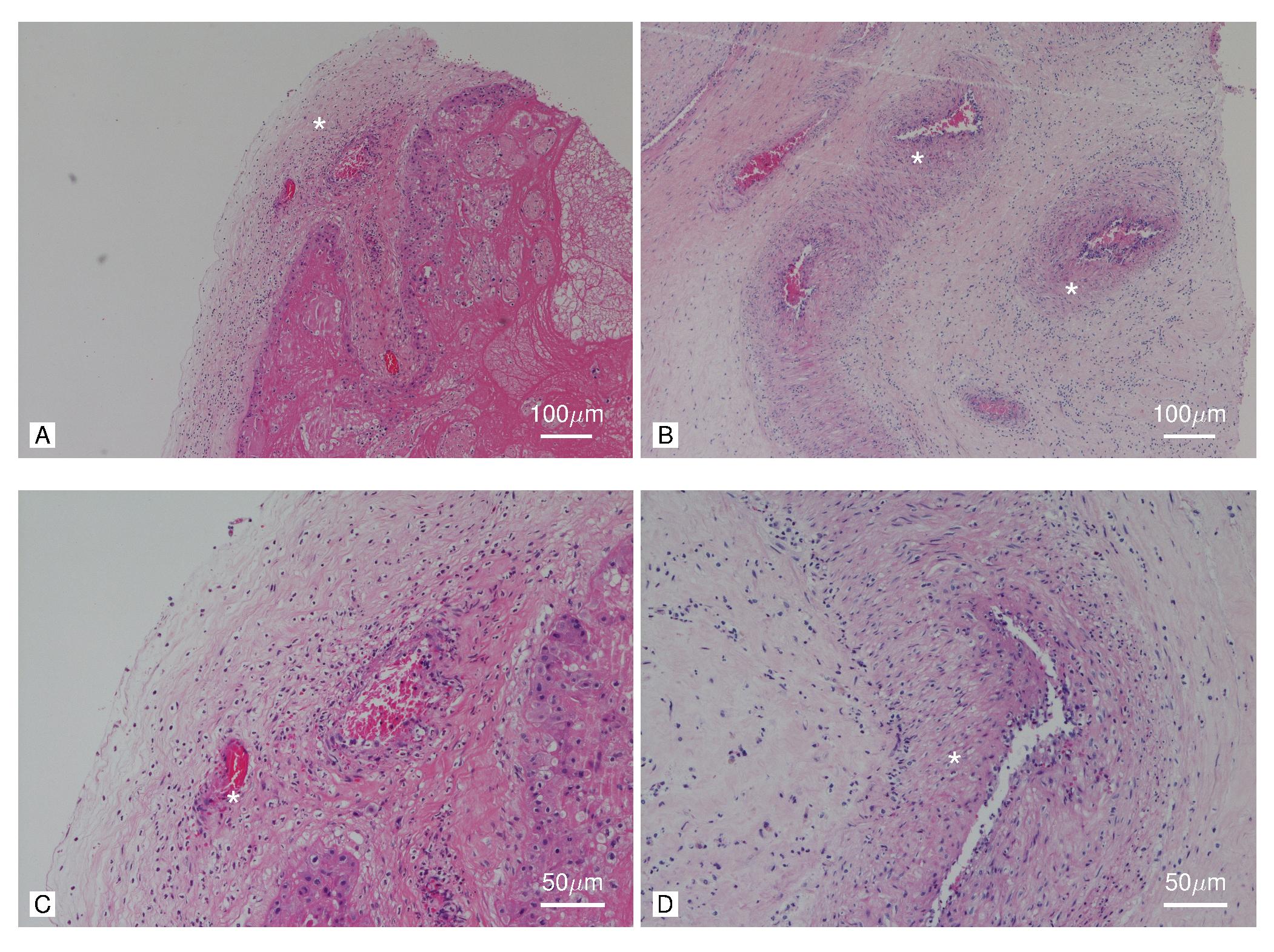

Supplement: Supplementary file 3 — Supplementary Figure S3. [file 41598_2021_3655_MOESM3_ESM.jpg]
