# Supplementary material for: Placental acute inflammation infiltrates and pregnancy outcomes: a retrospective cohort study
Source: Sci Rep. 2021 Dec 17;11:24165. doi: 10.1038/s41598-021-03655-4 (PMC8683439; doi:10.1038/s41598-021-03655-4)
Supplement: Supplementary file 4 — Supplementary Legends. [file 41598_2021_3655_MOESM4_ESM.docx]

**Supplemental material legends**

**Figure Legends**

**Supplemental Figure 1-** Funisitis, 4x magnification. A. Stage 1 grade 1, mild acute inflammatory infiltrate (*) in the umbilical vein. B. Stage 2 grade 1, mild acute inflammatory (*) in the umbilical artery. C. Stage 2 grade 2, severe acute inflammatory infiltrate (*) in the umbilical artery. D. Stage 3 grade 2, umbilical vessels concentric vasculitis (*).

**Supplemental Figure 2-** Chorioamnionitis, 10x magnification. A. Stage 1 grade 1, mild acute inflammatory infiltrate (*) in the chorion and decidua. B. Stage 1 grade 2, severe acute inflammatory infiltrate (*) in the chorion and decidua. C. Stage 2 grade 2 severe acute inflammatory infiltrate (*) in chorionic connective tissue and amnion. D. Stage 3 grade 2, necrotizing chorioamnionitis (*).

**Supplemental Figure 3-** Chorionic vasculitis - the images show the presence of acute inflammatory infiltrate in the wall of the vessels of the chorionic plate. A, B. 4x magnification. C, D. 10x magnification.

**Table Legends**

**Supplemental Table 1-** Population characteristics divided according to the presence or absence of funisitis.

**Supplemental Table 2-** Population characteristics divided according to the presence or absence of chorioamnionitis.

**Supplemental Table 3-** Population characteristics divided according to the presence or absence of chorionic vasculitis.
