# Supplementary material for: Placental acute inflammation infiltrates and pregnancy outcomes: a retrospective cohort study
Source: Sci Rep. 2021 Dec 17;11:24165. doi: 10.1038/s41598-021-03655-4 (PMC8683439; doi:10.1038/s41598-021-03655-4)
Supplement: Supplementary file 5 — Supplementary Tables. [file 41598_2021_3655_MOESM5_ESM.docx]

**Supplemental Table 1**- Population characteristics divided according to the presence or absence of funisitis.

|  | Funisitis absent (1592) | Funisitis present (178) | p |
| --- | --- | --- | --- |
| Maternal age (years old) | 33.00 (29.00-37.00) | 32.50 (28.00-37.00) | 0.604 |
| Maternal age > 40 years old | 11.49% (183/1592) | 10.11% (18/178) | 0.581 |
| Nulliparous | 59.80% (952/1592) | 65.73% (117/178) | 0.125 |
| Mode of conception |  |  |  |
| Spontaneous | 95.73% (1524/1592) | 94.38% (168/178) | 0.410 |
| Ovulation induction /IUI | 0.88% (14/1592) | 0.56% (1/178) | 0.660 |
| IVF/ICSI | 3.39% (54/1592) | 5.06% (9/178) | 0.260 |
| Gestational age at delivery (weeks) | 38.00 (37.00-40.00) | 39.00 (34.00-40.00) | 0.121 |
| Delivery <32 weeks | 6.03% (96/1592) | 20.79% (37/178) | <0.05 |
| Delivery <34 weeks | 11.37% (181/1592) | 24.16% (43/178) | <0.05 |
| Delivery <37 weeks | 24.31% (387/1592) | 27.53% (49/178) | 0.345 |
| Macro-regions of origin |  |  |  |
| Italy | 71.86% (1144/1592) | 63.48% (113/178) | <0.05 |
| East Europa | 14.13% (225/1592) | 14.04% (25/178) | 0.974 |
| Sub-Saharian Africa | 5.84% (93/1592) | 10.11% (18/178) | <0.05 |
| Arabian Countries | 2.89% (46/1592) | 4.49% (8/178) | 0.238 |
| Asia | 3.45% (55/1592) | 5.06% (9/178) | 0.278 |
| Others | 1.82% (29/1592) | 2.81% (5/178) | 0.363 |
| PRHDs | 7.47% (119/1592) | 1.69% (3/178) | <0.05 |
| Mode of labor |  |  |  |
| Spontaneous | 34.63% (551/1591) | 43.26% (77/178) | <0.05 |
| Induced | 37.21% (592/1591) | 40.45% (72/178) | 0.397 |
| Without labor | 28.16% (448/1591) | 16.29% (29/178) | <0.05 |
| Mode of delivery |  |  |  |
| Operative vaginal delivery | 9.17% (146/1592) | 10.11% (18/178) | 0.681 |
| Spontaneous | 43.59% (694/1592) | 41.01% (73/178) | 0.510 |
| Cesarean section | 47.24% (752/1592) | 48.88% (87/178) | 0.678 |

**Supplemental Table 2**- Population characteristics divided according to the presence or absence of chorioamnionitis.

|  | Chorioamnionitis absent (1435) | Chorioamnionitis present (335) | p |
| --- | --- | --- | --- |
| Maternal age (years old) | 33.00 (29.00-37.00) | 32.00 (28.00-36.00) | <0.05 |
| Maternal age > 40 years old | 11.85% (170/1435) | 9.25% (31/335) | 0.178 |
| Nulliparous | 59.58% (855/1435) | 63.88% (214/335) | 0.147 |
| Mode of conception |  |  |  |
| Spontaneous | 95.68% (1373/1435) | 95.22% (319/335) | 0.720 |
| Ovulation induction/IUI | 0.77% (11/1435) | 1.19% (4/335) | 0.440 |
| IVF/ICSI | 3.55% (51/1435) | 3.58% (12/335) | 0.980 |
| Gestational age at delivery (weeks) | 38.00 (37.00-39.00) | 39.00 (35.00-40.00) | 0.071 |
| Delivery <32 weeks | 5.30% (76/1435) | 17.01% (57/335) | <0.05 |
| Delivery <34 weeks | 10.73% (154/1435) | 20.90% (70/335) | <0.05 |
| Delivery <37 weeks | 24.11% (346/1435) | 26.87% (90/335) | 0.292 |
| Macro-regions of origin |  |  |  |
| Italy | 73.73% (1058/1435) | 59.40% (199/335) | <0.05 |
| East Europa | 12.68% (182/1435) | 20.30% (68/335) | <0.05 |
| Sub-Saharian Africa | 5.71% (82/1435) | 8.66% (29/335) | <0.05 |
| Arabian Countries | 2.79% (40/1435) | 4.18% (14/335) | 0.182 |
| Asia | 3.41% (49/1435) | 4.48% (15/335) | 0.348 |
| Others | 1.67% (24/1435) | 2.99% (10/335) | 0.115 |
| PRHDs | 7.80% (112/1435) | 2.99% (10/335) | <0.05 |
| Mode of onset of labor |  |  |  |
| Spontaneous | 33.68% (483/1434) | 43.28% (145/335) | <0.05 |
| Induced | 36.75% (527/1434) | 40.90% (137/335) | 0.158 |
| Without labor | 29.57% (424/1434) | 15.82% (53/335) | <0.05 |
| Mode of delivery |  |  |  |
| Operative vaginal delivery | 8.78% (126/1435) | 11.34% (38/335) | 0.145 |
| Spontaneous | 43.00% (617/1435) | 44.78% (150/335) | 0.554 |
| Cesarean section | 48.22% (692/1435) | 43.88% (147/335) | 0.15 |

**Supplemental Table 3**- Population characteristics divided according to the presence or absence of chorionic vasculitis.

|  | Chorionic vasculitis absent (1722) | Chorionic vasculitis present (48) | p |
| --- | --- | --- | --- |
| Maternal age (years old) | 33.00 (29.00-37.00) | 32.50 (26.00-36.00) | 0.355 |
| Maternal age > 40 years old | 11.38% (196/1722) | 10.42% (5/48) | 0.835 |
| Nulliparous | 60.05% (1034/1722) | 72.92% (35/48) | 0.072 |
| Mode of conception |  |  |  |
| Spontaneous | 95.59% (1646/1722) | 95.83% (46/48) | 0.940 |
| Ovulation induction/IUI | 0.87% (15/1722) | 0.00% (0/48) | 0.520 |
| IVF/ICSI | 3.54% (61/1722) | 4.17% (2/48) | 0.820 |
| Gestational age at delivery (weeks) | 38.00 (37.00-40.00) | 38.00 (35.75-40.00) | 0.663 |
| Delivery <32 weeks | 7.20% (124/1722) | 18.75% (9/48) | <0.05 |
| Delivery <34 weeks | 12.43% (214/1722) | 20.83% (10/48) | 0.084 |
| Delivery <37 weeks | 24.51% (422/1722) | 29.17% (14/48) | 0.460 |
| Macro-regions of origin |  |  |  |
| Italy | 71.25% (1227/1722) | 62.50% (30/48) | 0.187 |
| East Europa | 14.00% (241/1722) | 18.75% (9/48) | 0.351 |
| Sub-Saharian Africa | 6.21% (107/1722) | 8.33% (4/48) | 0.550 |
| Arabian Countries | 3.14% (54/1722) | 0.00% (0/48) | 0.213 |
| Asia | 3.48% (60/1722) | 8.33% (4/48) | 0.076 |
| Others | 1.92% (33/1722) | 2.08% (1/48) | 0.934 |
| PRHDs | 7.03% (121/1722) | 2.08% (1/48) | 0.182 |
| Mode of onset of labor |  |  |  |
| Spontaneous | 34.86% (600/1721) | 58.33% (28/48) | <0.05 |
| Induced | 37.71% (649/1721) | 31.25% (15/48) | 0.362 |
| Without labor | 27.43% (472/1721) | 10.42% (5/48) | <0.05 |
| Mode of delivery |  |  |  |
| Operative vaginal delivery | 9.29% (160/1722) | 8.33% (4/48) | 0.821 |
| Spontaneous | 43.32% (746/1722) | 43.75% (21/48) | 0.953 |
| Cesarean section | 47.39% (816/1722) | 47.92% (23/48) | 0.942 |
